# Central metabolism and development are rewired in lichenized cyanobacteria

**DOI:** 10.1093/ismejo/wraf166

**Published:** 2025-08-06

**Authors:** Diego Garfias-Gallegos, Carlos J Pardo-De la Hoz, Diane L Haughland, Nicolas Magain, Blanka Aguero, Jolanta Miadlikowska, François Lutzoni

**Affiliations:** Department of Biology, Duke University, Durham, NC 27708, United States; Department of Biology, Duke University, Durham, NC 27708, United States; Department of Renewable Resources, Faculty of Agricultural, Life & Environmental Sciences, University of Alberta, Edmonton, AB T6G 2H1, Canada; Evolution and Conservation Biology, InBioS Research Center, Université de Liège, Liège 4000, Belgium; Department of Biology, Duke University, Durham, NC 27708, United States; Department of Biology, Duke University, Durham, NC 27708, United States; Department of Biology, Duke University, Durham, NC 27708, United States

**Keywords:** biological nitrogen fixation, boreal forest, feathermoss, glycogen metabolism, lichen symbiosis, molybdenum nitrogenase, Nostoc, photoautotrophy, symbiotic nutrient exchange, vanadium nitrogenase

## Abstract

*Nostoc* cyanobacteria are among the few organisms capable of fixing both carbon and nitrogen. These metabolic features are essential for the cyanolichen symbiosis, where *Nostoc* supplies both carbon (as glucose) and nitrogen (as ammonium) to a cyanolichen-forming fungal partner. This nutrient flow was established by seminal biochemical studies published in the 20^th^ century. Since then, cyanolichen metabolism has received little attention, and the molecular mechanisms that underlie the physiology of lichenized *Nostoc* remain mostly unknown. Here, we aimed to elucidate the genomic and transcriptional changes that enable *Nostoc’*s metabolic role in cyanolichens. We used comparative genomics across 243 genomes of *Nostoc s. lat.* coupled with metatranscriptomic experiments using *Peltigera* cyanolichens. We found that genes for photoautotrophic carbon fixation are upregulated in lichenized *Nostoc.* This likely results in a higher rate of carbon fixation that allows *Nostoc* to provide carbon to the fungal partner while meeting its own metabolic needs. We also found that the transfer of ammonium from *Nostoc* to the lichen-forming fungus is facilitated by two molecular mechanisms: (i) transcriptional downregulation of glutamine synthetase, the key enzyme responsible for ammonium assimilation in *Nostoc*; and (ii) frequent losses of a putative high-affinity ammonium permease, which likely reduces *Nostoc’*s capacity to recapture leaked ammonium. Finally, we found that the development of motile hormogonia is downregulated in lichenized *Nostoc*, which resembles the repression of motility in *Nostoc* symbionts after they colonize symbiotic cavities of their plant hosts. Our results pave the way for a revival of cyanolichen ecophysiology in the omics era.

## Introduction

Cyanobacteria from the genus *Nostoc* are some of the most versatile microorganisms on Earth. They possess a rare combination of metabolic traits, including the capacity to fix atmospheric nitrogen and the ability to obtain nutrients both heterotrophically and photoautotrophically [1–3]. They occur as multicellular colonies with cells that can differentiate into three different cell types, including nitrogen-fixing heterocysts and motile hormogonia [4, 5]. This metabolic and developmental versatility has allowed *Nostoc* to colonize a myriad of terrestrial and aquatic ecosystems, from the tropics to polar regions, and thrive in both free-living and symbiotic lifestyles [6, 7].

Many *Nostoc* lineages form symbioses with plants (hornworts, liverworts, mosses, *Azolla* ferns, cycads, and *Gunnera* angiosperms) and fungi (the glomeromycete *Geosiphon*, and most cyanolichen-forming fungi e.g. from the order Peltigerales) [8–11]. In symbiosis with plants, *Nostoc* releases fixed nitrogen to the plant host while the plant transfers fixed organic carbon to the *Nostoc* symbiont [12]. The establishment and metabolic activity of these symbioses rely on *Nostoc* cellular differentiation modulated by chemical cues secreted by the plant hosts, which can induce or repress the development of motile hormogonia (enabling the colonization of plant tissues) and nitrogen-fixing heterocysts [8]. Conversely, when in symbiosis with fungi (e.g. cyanolichens), *Nostoc* transfers both nitrogen and fixed organic carbon to the fungus [13, 14], providing the majority of the fungus’s elementary macronutrient input.

The symbiotic interactions that *Nostoc* forms with mosses and cyanolichen fungi are important for global nutrient cycling because they account for the majority of biological nitrogen fixation in high-latitude ecosystems such as boreal and arctic biomes [15–18]. In the context of climatic and chemo-atmospheric changes, it is increasingly important to understand the biochemical, genomic, and regulatory shifts that enable *Nostoc* to fulfill its functional contributions to ecosystems. This has been studied extensively for *Nostoc* in symbiosis with plants, including mosses [8, 12, 19–22]. However, this is not the case for cyanolichens, especially since seminal studies from the 20^th^ century revealed key biochemical aspects of the flow of nitrogen and carbon from *Nostoc* to its fungal partners [13, 14, 23–26]. As a result, the genomic and regulatory underpinnings of the metabolic dynamic in lichenized *Nostoc* remain mostly unknown.

In this study, we aimed to elucidate the genomic and transcriptional changes that enable *Nostoc’*s metabolic role in cyanolichens. We used comparative genomics and metatranscriptomic experiments focused on pathways for carbon and nitrogen metabolism as well as hormogonia and heterocyst development. We compiled a dataset of 243 genomes representing the diversity of *Nostoc s. lat.*, including 146 genomes of lichenized *Nostoc* (18 of which were sequenced as part of this study; Supplementary Table S1). We conducted metatranscriptomic experiments with *Peltigera* cyanolichens, a genus that has been the main model for cyanolichen physiological and transcriptomic studies, and which includes some of the most abundant cyanolichens in boreal and arctic biomes [6, 27–30]. *Peltigera* cyanolichens often grow on feathermosses, which also host epiphytic *Nostoc* symbionts [18]. This provided an ideal setting to study the transcriptomics of lichenized *Nostoc* in field conditions because we could compare the expression profiles of lichenized *Nostoc* to epiphytic *Nostoc* symbionts of feathermosses adjacent to cyanolichen thalli. Using these approaches, we identified key genomic and transcriptional changes indicative of the metabolic and developmental rewiring associated with the lichenized lifestyle in *Nostoc*.

## Materials and methods

### Lichen and feathermoss substrate sampling

We conducted our sampling at a boreal forest site in Alberta, Canada (55°14′35.5″N, 114°02′15.3″W, Supplementary Fig. S1A and B). We selected this site because *Peltigera* species that interact with the four most common *Nostoc* sections in Alberta cooccur in this area [6]. In the field, *Peltigera* thalli showed variability in water content within and between thalli. This variation is expected because lichens equilibrate with the moisture of the surrounding environment [13, 14]. To account for this variability, we collected two sets of samples for our metatranscriptomic experiment: one from the lichen specimens and their surrounding moss substrates in natural conditions under the forest canopy (natural), and another from the same lichen specimens and moss substrates but after removing them from the site and saturating them with water under natural light exposure (water-saturated; Supplementary methods; Supplementary Tables S2 and S3). The parallel experiment with water-saturated samples allowed us to ensure that our results were robust when water content and light conditions were homogeneous across samples. Overall, we collected 24 samples for each set (i.e. 12 from lichen thalli and 12 from the feathermoss substrates) for a total of 48 samples across the natural and water-saturated sets. All lichen and feathermoss samples were immediately flash-frozen in a liquid nitrogen dry shipper, shipped in dry ice, and stored at −80°C until nucleic acid isolation (Supplementary methods).

### Newly generated genomes of *Nostoc*

We recovered 13 *Nostoc* metagenome-assembled genomes (MAGs) from the 12 lichen libraries, and six MAGs from the 12 feathermoss libraries (Supplementary methods). After removing contaminant contigs from the MAGs (Supplementary methods), we retained 12 *Nostoc* MAGs from the lichen libraries (one from each lichen thallus), and six MAGs from the feathermoss libraries with >90% completeness and < 5% contamination (Supplementary Table S1).

### Sampling of publicly available genomes of *Nostoc s. lat.*

We retrieved 225 genomes of *Nostoc s. lat.* from public databases (Supplementary Table S1). This includes *Nostoc s. str.* and closely related genera *Desmonostoc* and *Komarekiella*. These genera form a monophyletic group, which used to be classified as *Nostoc* [6, 31, 32]*.* We also included the genomes of *Anabaena cylindrica* PCC 7122, *Aphanizomenon flos-aquae* NIES-81, and *Cylindrospermum stagnale* PCC 7417 as outgroup taxa for phylogenetic analyses [6, 33, 34]. Overall, our genomic sampling included 243 genomes of *Nostoc s. lat.* (225 publicly available and 18 MAGs sequenced in this study) and three outgroup taxa, for a total of 246 genomes (Supplementary Table S1).

### Phylogenetic analyses

To infer a species tree of *Nostoc s. lat.*, we used a phylogenetic inference pipeline described in a previous study [6]. We extracted 1517 BUSCO markers from the 246 genomes (Supplementary Table S1), aligned nucleotide sequences using MAFFT v7.475 [35] and PAL2NAL v14 [36], and inferred maximum likelihood gene trees with IQ-Tree v1.6.12 [37]. Then, we used the resulting gene trees as input to infer a species tree with weighted-ASTRAL [38]. We used the weighted-ASTRAL tree to classify the genomes according to the phylogenetic framework proposed in [6]. We also inferred a protein family tree for ammonium permeases to understand their evolution and diversity in *Nostoc s. lat.* (Supplementary methods).

### Genome annotation

We used two approaches for genome annotation. The first approach relied on automatic annotation software. We predicted open-reading frames (ORFs) in the 246 genomes using Prokka v1.14.5 [39]. The resulting gbk and fasta files were used as input for DRAM v1.5.0 [40], which used the PFAM, UniRef90, RefSeq, CAZyDB, and KEGG v110.0 databases (downloaded on October 23^rd^, 2023) to search for primary and secondary metabolism genes. We used the distill option in DRAM to annotate transfer ribonucleic acid (tRNA) and ribosomal RNA (rRNA) sequences. The second approach consisted of a targeted search for specific genes (Supplementary Table S4; Supplementary methods) involved in metabolic and developmental pathways in *Nostoc s. lat.* (including C and N metabolism as well as hormogonia and heterocyst development), which are not typically annotated by automated pipelines.

### Metatranscriptomic read filtering and mapping

Paired-end metatranscriptomic reads were merged, quality-checked, and filtered using fastp v0.23.4 [41]. We trimmed reads in a sliding window of 15 bp when <80% bases had < Q18 PHRED score and retained trimmed reads with >100 bp. Then, we extracted the *Nostoc* RNA reads from each metatranscriptomic library using our custom kraken2-bracken database (Supplementary methods). We removed rRNA sequences by mapping the extracted *Nostoc* reads to the rRNA sequences annotated by DRAM.

We used bwa v0.7.18 and samtools v1.21 [42, 43] to map the extracted reads of *Nostoc* from each metatranscriptomic library to all annotated genes. However, we used different reference genomes to map the reads from each library because the *Nostoc* strains in the lichen and feathermoss samples belonged to different lineages (Supplementary Fig. S2; Supplementary methods). Following documentation from DESeq2 and edgeR [44, 45], we only kept the libraries whose total median mapped-reads value was above zero for the genes present in all the reference genomes. After this filtering, we retained mapping data for 24 libraries of lichenized *Nostoc* (12 natural and 12 water-saturated) and 13 libraries of feathermoss-associated *Nostoc* (six natural and seven water-saturated). We used the mapping results to generate feature counts tables using CoverM v0.7.8 (https://github.com/wwood/CoverM).

### Differential expression analysis

Read abundance was first normalized using the Trimmed Mean of M-values normalization method from the R package edgeR v4.2.1 [45]. We then used the normalized data as input for DESeq2 v1.44.0 [44] to test differential expression across the lifestyle parameter, i.e. lichenized vs. feathermoss-associated, with α = 0.05. The differential expression analyses were performed separately for natural and water-saturated treatments. For each treatment, we compared the expression from all lichenized libraries with the expression of all feathermoss-associated samples (i.e. 12 lichenized against six feathermoss-associated, for natural conditions, and 12 lichenized against seven feathermoss-associated for water-saturated samples). We visualized the resulting Log_2_ Fold Change (Log_2_FC) and adjusted *P* values using ggplot2 v3.5.1 [46].

## Results and discussion

### Lichenized and moss-associated *Nostoc* strains belong to five phylogenetic sections

We found that the 18 *Nostoc* MAGs we generated belong to five different *Nostoc* sections (i.e. infrageneric lineages that include multiple closely related species-level clades [6]; Supplementary Fig. S2). The 12 lichenized strains are part of section 3.1 (*P. canina s. lat.* c1–c3), section 3.5 (*P. appalachiensis* n1–n3), section 3.6 (*P. elisabethae* e1–e3), and section 2.4 (*P. malacea* m1–m3). Five of the six moss-associated *Nostoc* strains belong to section 2.4, and one is part of section 3.7 (Supplementary Fig. S2). Our differential expression analyses focused on orthologous genes conserved across these lineages of *Nostoc*, as done previously [21].

### Genes for photosynthetic carbon fixation are upregulated in lichenized *Nostoc*

**Figure 1:**
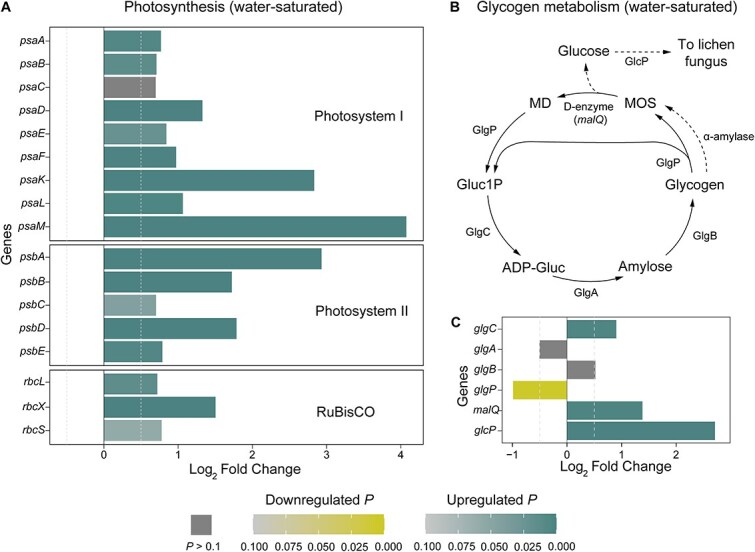
Photosynthesis and putative symbiotic glycogen degradation are upregulated in lichenized *Nostoc* in water-saturated samples. (A) Differential expression of genes that encode three key protein complexes involved in photosynthetic carbon fixation. (B) Glycogen synthesis and degradation in cyanobacteria. Solid arrows show the canonical pathway (https://www.genome.jp/pathway/hsa00500) [116]. Dashed arrows show the proposed mechanism of glycogen degradation that leads to glucose released by lichenized *Nostoc* [26]. (C) Differential expression of glycogen metabolism genes. Log_2_ Fold Change and adjusted *P* values were estimated with DESeq2 based on 12 lichenized and seven moss-associated *Nostoc* RNA-seq libraries from water-saturated samples. Gene descriptions are in Supplementary Table S4. Gluc1P: glucose-1-phosphate; ADP-Gluc: adenosine diphosphate glucose; MOS: maltooligosaccharides; MD: maltodextrins; GlgC: glucose-1-phosphate adenylyltransferase; GlgA: glycogen synthase; GlgB: glucan-branching enzyme; GlgP: glycogen phosphorylase; *malQ*: putative D-enzyme; GlcP: glucose permease.

We found that almost all genes that code for three key protein complexes involved in photosynthetic carbon fixation (photosystem I [PSI], photosystem II [PSII], and RuBisCO) were significantly upregulated (Log_2_FC > 0.5 and *P <* 0.05; Supplementary Table S4) in lichenized *Nostoc* from water-saturated (Fig. 1A) and natural samples (Supplementary Fig. S3). In addition, the genes encoding seven of the nine additional enzymes from the Calvin-Benson cycle for CO_2_ fixation (*pgk*, *gapA*, *fbaA*, *fbaB*, *tktA*, *prkB*, and *talA* [in natural samples only]) were also upregulated (Supplementary Fig. S4). These results suggest that lichenized *Nostoc* have higher photosynthetic activity compared to *Nostoc* associated with feathermosses.

Genes that encode the PSI, PSII, and RuBisCO protein complexes were still among the top 12% most abundant transcripts in *Nostoc* associated with feathermosses. This supports the view that *Nostoc* is fixing carbon when associated with feathermosses despite being able to assimilate organic carbon fixed by the moss [19, 21]. In contrast, lichenized *Nostoc* transfer 40%–75% of their fixed carbon to the cyanolichen fungus and must still meet the carbon requirements to survive and grow in the thallus [13, 24, 28, 47]. As expected, based on the high carbon demand from the fungus, we did not observe significant changes in the expression of most genes from cellular respiration pathways (i.e. glycolysis, citrate cycle, and oxidative phosphorylation) in lichenized *Nostoc.* The exception was genes of the NAD(P)H:quinone oxidoreductase and the cytochrome b6-f complexes (Supplementary Fig. S5), which participate in both photosynthetic electron transport and oxidative phosphorylation during respiration. This supports the hypothesis that photosynthesis is upregulated in lichenized *Nostoc* (Fig. 1, Supplementary Figs S3 and S4), which results in excess fixed carbon that is transferred to the cyanolichen fungus rather than used for *Nostoc*’s energetic demands.

What remains unclear is the identity of the signals that trigger the transcriptional upregulation of photosynthesis genes in cyanolichens. One possibility is that the transfer of carbon from *Nostoc* to the lichen fungus alters the carbon balance in the *Nostoc* cells. Changes in carbon availability regulate the expression of photosynthesis and carbon fixation genes in plants, green algae, and cyanobacteria [48–50]. For example, inducing sucrose release in *Synechococcus elongatus* upregulates RuBisCO genes and enhances photosynthetic efficiency [51–53]. This resembles the release of glucose by lichenized *Nostoc*, which is the mechanism of carbon transfer to the lichen fungus [13, 24, 54]. The rate of glucose release declines sharply after *Nostoc* is isolated from the lichen thallus [26]. This suggests that the release of glucose from *Nostoc* is regulated by symbiosis-specific signals [25, 26] that may alter the carbon balance in the cells, leading to transcriptional upregulation of photosynthetic carbon fixation genes (Fig. 1, Supplementary Figs S3 and S4).

### *malQ* and *glcP* are likely involved in glucose release from lichenized *Nostoc*

Previous studies found strong evidence that the glucose released from lichenized *Nostoc* is derived from intracellular glycogen pools [13, 24, 25, 55]. A former study [26] proposed that glycogen is first broken down by an α-amylase (EC 3.2.1.1) into maltooligosaccharides, which are then disproportionated by a D-enzyme (disproportionating transglycosylase; EC 2.4.1.25) into free glucose and maltodextrins (Fig. 1B). The free glucose product of this reaction would then move to the lichen fungus mediated by a passive glucose channel (Fig. 1B). We found that all 243 genomes of *Nostoc s. lat.* in our sampling contain a homolog of the *malQ* gene (Supplementary Table S5), which encodes a putative D-enzyme [56, 57]. In addition, we found a single putative glucose permease gene (*glcP* homolog; Supplementary methods) in all 146 genomes of lichenized *Nostoc* (Supplementary Table S5). Both *malQ* (Log_2_FC = 1.38, *P* = 0.001) and *glcP* (Log_2_FC = 2.71, *P* = 3.5 × 10^−5^) were upregulated in lichenized *Nostoc* (Fig. 1C). An upregulated *malQ* signal is consistent with previous observations [26] that the D-enzyme activity was significantly higher in the freshly isolated *Nostoc* symbiont of *P. horizontalis* compared to the *Nostoc* in pure culture*.* Moreover, *glcP* plays an essential role in the establishment of the symbiosis between *Nostoc* and *Anthoceros* [58]. Therefore, our results suggest that *malQ* and *glcP* play a key role in glucose transfer from *Nostoc* to the cyanolichen fungus [26].

In non-lichenized conditions, D-enzymes participate in glycogen degradation by disproportionating short maltooligosaccharides to form maltodextrins of sufficient length that can be further degraded by glycogen phosphorylase (GlgP; encoded by *glgP*; Fig. 1B) [56, 59, 60]. However, we found that *glgP* was downregulated in lichenized *Nostoc* (Log_2_FC = −0.98, *P* = 7.9 × 10^−4^; Fig. 1C). This further indicates that the transcriptional upregulation of *malQ* in lichenized *Nostoc* is unrelated to the canonical degradation of glycogen and may instead be associated with symbiotic carbon transfer.

Only 112 of the 146 genomes of lichenized *Nostoc s. lat.* in our sampling contain a homolog of the *amy1* gene (Supplementary Table S5) that encodes a known α-amylase in a strain of *Nostoc s. lat.* [61]. Glycogen may be broken down by a different enzyme with amylolytic activity as part of symbiotic carbon transfer in cyanolichens [62]. We were not able to compare the expression of *amy1* because the gene was missing from five of the twelve lichenized *Nostoc* strains included in the metatranscriptomic assay (Supplementary Table S5). However, unlike the D-enzyme, a previous study did not find differential amylase activity between the freshly isolated *Nostoc* symbiont compared to the *Nostoc* in pure culture [26]. Thus, the amylolytic enzyme is not expected to be differentially expressed.

### Heterocyst differentiation is downregulated in lichenized *Nostoc*

**Figure 2:**
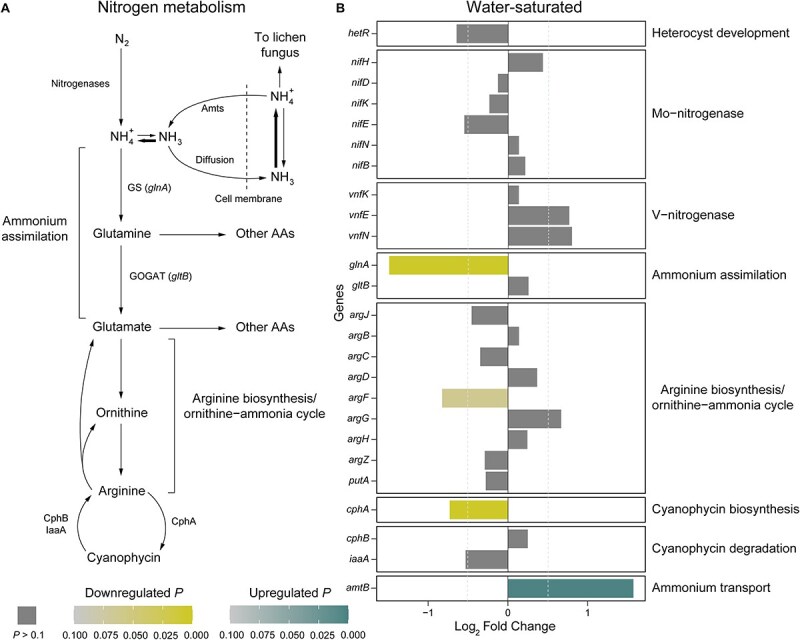
Downregulated ammonium assimilation is associated with signatures of growth under nitrogen-deficient conditions in lichenized *Nostoc*. (A) Summary of dinitrogen assimilation pathway in cyanobacteria [78, 84, 117–119]. (B) Differential expression of genes involved in dinitrogen assimilation. Log_2_ Fold Change and adjusted *P* values were estimated with DESeq2 based on 12 lichenized and seven moss-associated *Nostoc* RNA-seq libraries from water-saturated samples. Gene descriptions are in Supplementary Table S4. Amts: ammonium transporters; GS: glutamine synthetase; AAs: amino acids; GOGAT: glutamine oxoglutarate aminotransferase (also known as glutamate synthase); CphA: cyanophycin synthetase; CphB: cyanophycinase; IaaA: isoaspartyl aminopeptidase.

In *Nostoc*, nitrogen fixation occurs in specialized cells called heterocysts, which create a microoxic environment required for the activity of nitrogenase enzymes [5]. We found that *hetR*, which encodes an essential regulator of many genes involved in heterocyst differentiation, was downregulated in lichenized *Nostoc* [63, 64]. This result was statistically significant only in the natural samples (Log_2_FC = −1.41, *P* = 7.8 × 10^−4^; Supplementary Fig. S6), but the trend was similar in the water-saturated samples (Log_2_FC = −0.64, *P* = 0.09; Fig. 2B). In addition, we found that other genes participating in the early steps of heterocyst differentiation (*pipY*, *sepF*, and *ftsZ*) [65, 66] were downregulated in lichenized *Nostoc* under natural and water-saturated conditions. However, this result was statistically significant only in water-saturated samples (Supplementary Table S4). This is consistent with previous studies that found lower heterocyst frequencies in *Nostoc* symbionts of bimembered *Peltigera* cyanolichens (2%–6%) compared to free-living *Nostoc* (5%–10%) [8, 67–69]. Conversely, heterocyst frequencies are expected to be similar between *Nostoc* associated with feathermosses compared to free-living *Nostoc* [21]. *Nostoc* cells can account for over a third of the biomass in *Peltigera* cyanolichens at a density of ~9 × 10^6^ cells per cm^2^ of thallus [14, 68]. This high cell density enables sufficient input of fixed nitrogen to the cyanolichen thallus despite the low frequency of heterocysts [14, 68].

### Vanadium-dependent nitrogenase is most common in lichenized *Nostoc*

Nitrogen fixation by *Nostoc* can be mediated by two types of nitrogenase enzymes: a molybdenum-dependent nitrogenase (Mo-Nase; encoded by *nif* genes), and a vanadium-dependent nitrogenase (V-Nase; encoded by *vnf* genes) [70, 71]. Three of the six *Nostoc* MAGs from feathermosses lacked a copy of *vnfDG*, which encodes an essential structural component of the V-Nase [70, 71]. Consequently, we were unable to compare the expression of *vnfDG* between lichenized *Nostoc* with their adjacent moss-associated *Nostoc*. Nevertheless, we detected expression but no significant differential expression of the remaining genes encoding both enzymes and their co-factors (*nifHDKENB* for Mo-Nase and *vnfKEN* for V-Nase; Fig. 2 and Supplementary Fig. S6).

**Figure 3:**
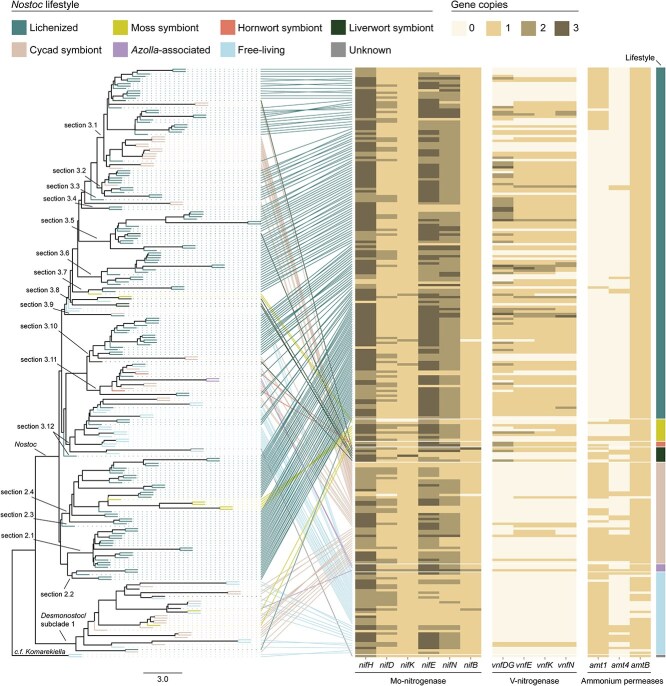
Comparative genomics of nitrogenase and ammonium permease genes across 243 genomes of *Nostoc s. lat.* Each row of the heatmap corresponds to one of the 243 genomes of *Nostoc s. lat*. The gene count matrix with associated metadata is in Supplementary Table S5. Lifestyle categories were assigned according to the source of isolation or sequencing of the *Nostoc* genomes or MAGs (Supplementary Table S1). The phylogenetic position of each genome is shown with lines connecting each row of the heatmap to a phylogenomic tree of *Nostoc* on the left. The tree was inferred with weighted-ASTRAL using 1517 genes. Branch lengths represent coalescent units. This is the same tree as in Supplementary Fig. S2 but without the outgroup taxa. Phylogenetic sections (2.1–3.12) were delimited as described previously [6].

In our larger sample of genomes, we found that 66% (97/146) of lichenized *Nostoc s. lat.* Have a full complement of *vnf* genes encoding V-Nase (Fig. 3). In addition, 53% (8/15) of the genomes from *Nostoc* symbionts of bryophytes (i.e. hornworts, liverworts, and mosses) also have a full complement of *vnf* genes (Fig. 3). In contrast, V-Nase is rare in genomes of free-living and cycad-symbiotic *Nostoc*, with only 6% (2/34) and 7% (3/42) of genomes having a full complement of *vnf,* respectively (Fig. 3). These results are robust even if we consider an estimated false negative rate of V-Nase gene detection of 11% in MAGs (see Supplementary methods). Thus, our findings support the view that V-Nase activity is highly advantageous in the cyanolichen and *Nostoc*-bryophyte symbioses [72–74]. Although Mo-Nase is typically responsible for most of the nitrogen input, the contribution of V-Nase can be substantial at low temperatures and when Mo availability is low [17, 75]. For example, Mo-Nase reactivity is higher than V-Nase at temperatures >15°C but V-Nase is as efficient as Mo-Nase at cooler temperatures, which are typical of boreal ecosystems [76]. Moreover, Mo is the scarcest micronutrient on the continental crust, and it is a limiting factor for nitrogen fixation in cryptogamic covers such as cyanolichens and bryophytes where metal availability depends on atmospheric deposition [77, 78]. Therefore, *Nostoc* symbionts capable of using both Mo- and V-Nases may be selected in these nitrogen-demanding symbioses, especially when living in cold environments where nitrogen and Mo can be limiting. In contrast, retaining the V-Nase genes may not be advantageous for free-living *Nostoc* given the absence of symbiotic demand for nitrogen. Similarly, cycad-symbiotic *Nostoc* may rarely experience Mo-limitation given the capacity of the plant host to acquire trace metals through the root system, which reduces the need for V-Nase activity.

### Ammonium assimilation in lichenized *Nostoc* is downregulated resembling gene expression associated with cellular nitrogen deficiency

Ammonium is the main product of nitrogen fixation and the preferred source of combined nitrogen for cyanobacteria [79] (Fig. 2A). In non-symbiotic conditions*,* the pathway of ammonium assimilation in *Nostoc* starts with the key enzyme glutamine synthetase (GS), which uses ammonium and glutamate to produce glutamine [79] (Fig. 2A). In symbioses with hornworts and cyanolichens, the activity of *Nostoc*’s GS is significantly lower than in a free-living state [12, 14, 23, 80–82]. Because of this, the non-assimilated ammonium leaks from the cells and is then taken up by the plant or fungal host [14, 23, 82, 83] (Fig. 2A). In contrast, epiphytic *Nostoc* symbionts of feathermosses are thought to release organic nitrogen rather than ammonium, and their GS expression level is similar to non-symbiotic *Nostoc* [9, 21]. We found that *glnA*, which encodes GS, was downregulated in lichenized *Nostoc* compared to *Nostoc* associated with feathermosses (Fig. 2B and Supplementary Fig. S6). This would be a different regulatory mechanism than in *Nostoc* symbionts of hornworts, where GS synthesis is not impaired, and the lower activity of GS is likely due to posttranslational modifications [80, 84].

We found that *cphA*, which encodes the enzyme responsible for the biosynthesis of cyanophycin (Fig. 2A), was downregulated in lichenized *Nostoc* (Fig. 2B and Supplementary Fig. S6). Cyanophycin is a co-polymer of arginine and aspartate that serves as a nitrogen reservoir in cyanobacteria [85, 86]. This result is consistent with previous reports that lichenized *Nostoc* have a substantially lower frequency of cyanophycin granules compared to free-living *Nostoc* [82, 87]. Our study also revealed that *amtB*, which encodes a putative ammonium permease [88, 89], was upregulated in lichenized *Nostoc* (Fig. 2B and Supplementary Fig. S6). Both downregulated *cphA* and upregulated *amtB* are typically associated with growth under nitrogen-deficient conditions [90, 91]. Therefore, our results suggest that lichenized *Nostoc* are in a regulatory state that resembles nitrogen deficiency. This could be a direct result of decreased nitrogen input due to genes involved with ammonium assimilation being downregulated (Fig. 2B and Supplementary Fig. S6). Alternatively, a decrease in the biosynthesis of cyanophycin may enable the transfer of nitrogen to the cyanolichen fungus directly. This could be because of the known role of this polymer in buffering the movement of fixed nitrogen between heterocysts and vegetative cells of *Nostoc* [86].

### Loss of a high-affinity ammonium permease may facilitate ammonium transfer in cyanolichens

We found that the genomes of *Nostoc s. lat.* encode up to three types of putative ammonium permeases from the AMT/MEP/Rh family (Supplementary Table S6) [92]: Amt1, Amt4, and AmtB (Supplementary Fig. S7). Each type is closely related to known ammonium permeases from *Anabaena* sp. PCC 7120 (Amt1, Amt4, and AmtB) [89, 93] and *Synechococcus elongatus* PCC 7942 (Amt1 and AmtB) (Supplementary Fig. S7) [88, 94]. In both strains, Amt1 is responsible for ~95% of ammonium uptake, particularly when the concentration of extracellular ammonium is low [79, 89, 94]. This plays an important role in recapturing ammonia leaked from cyanobacterial cells [89, 94]. We found that 82% (120/146) of lichenized *Nostoc s. lat.* lack *amt1* (Fig. 3)*.* In contrast, 97% (33/34) of free-living *Nostoc s. lat.* have one *amt1* (Fig. 3). In addition, 78% (33/42) of *Nostoc s. lat.* symbionts of cycads, which transfer organic nitrogen instead of ammonium to their hosts [12, 95], also have one *amt1* (Fig. 3). A logistic regression that accounts for the underlying phylogeny (Supplementary methods) indicated that the lichenized lifestyle is associated with a 73% decrease in odds of *amt1* presence (β_lichenized_ = −1.307, odds ratio = *e*^ßlichenized^ = 0.27, *P* value = 0.006). The frequent absence of *amt1* in lichenized *Nostoc s. lat.* may be due to selection for strains with a reduced capacity to recapture leaked ammonia. This is similar to the repression of ammonium uptake in symbiotic rhizobia from legume root nodules [96, 97], and it might facilitate the transfer of fixed nitrogen from *Nostoc* to the cyanolichen fungus.

Nearly all *Nostoc s. lat.* genomes (96%, 233/243) we examined have one *amtB* (Fig. 3). This is surprising because the AmtB permease is not essential for ammonium uptake in model cyanobacteria strains [88]. Moreover, mutants with only AmtB take up ammonium at only ~3% of the rate of wild-type strains [94]. However, 53% (129/243) of *Nostoc s. lat.* genomes, including 78% (115/146) of lichenized *Nostoc*, have only *amtB* (Fig. 3). This suggests that AmtB has an important function other than ammonium uptake, as proposed for other nitrogen-fixing bacteria [9, 90]. For example, in *Rhodobacter capsulatus*, the AmtB permease can regulate nitrogenase activity by recruiting P_II_ signal transduction proteins even if the rate of ammonium transport is reduced [98, 99]. Furthermore, the formation of complexes between AmtB and P_II_ signal transduction proteins to regulate nitrogen metabolism is widespread across bacteria [100–102]. Therefore, AmtB may be part of the regulatory network of nitrogen metabolism in *Nostoc s. lat.*

### Development of motility structures is downregulated in lichenized *Nostoc*

**Figure 4:**
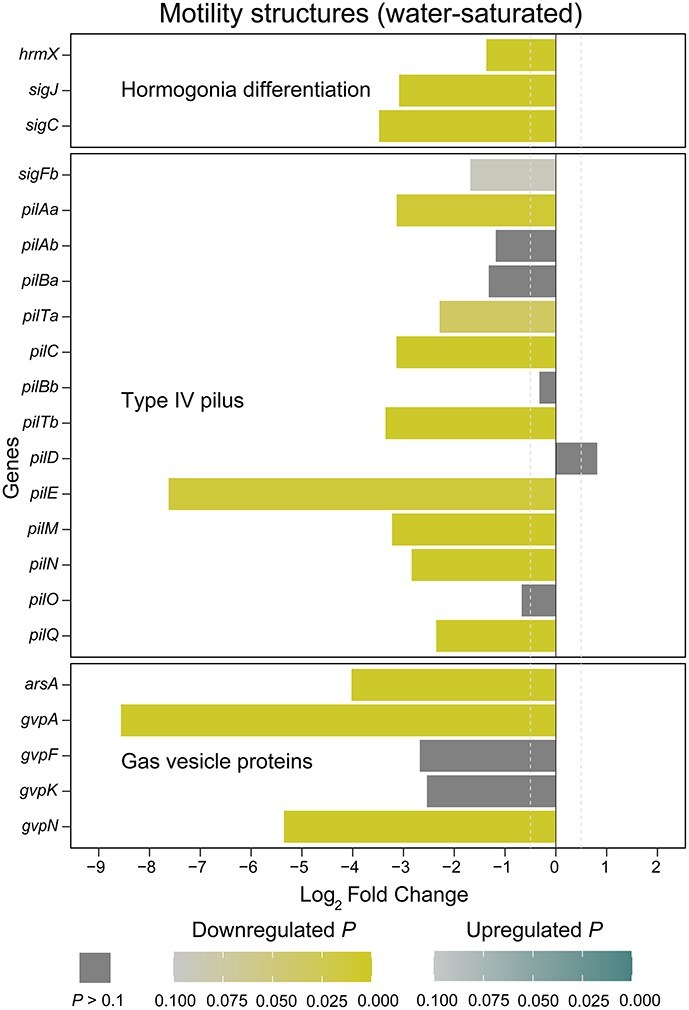
The development of motility structures is downregulated in lichenized *Nostoc*. Differential expression of structural and regulatory genes for the development of motility structures. Log_2_ Fold Change and adjusted *P* values were estimated with DESeq2 based on 12 lichenized and seven moss-associated *Nostoc* RNA-seq libraries from water-saturated samples. *hrmX* was initially called *hrmK* [103] and was later relabeled *hrmX* [4]. Gene descriptions are in Supplementary Table S4.

In epiphytic symbiosis with feathermosses, motility is not repressed, and *Nostoc* colonies contain a mixture of vegetative filaments with heterocysts and motile hormogonia [21]. In lichenized *Nostoc*, we found that several structural and regulatory genes involved in the development of motile hormogonia were downregulated (*hrmX*, *sigJC, pil*, and *gvp* genes; Fig. 4). The *hrmX* gene encodes a putative hybrid histidine kinase that acts as one of the master regulators of the early development of motile hormogonia [4, 103]. Similarly, the sigma factors encoded by *sigJ* and *sigC* control the expression of cell division and signal transduction mechanisms essential for the early development of hormogonium morphology [104]. The *pil* genes are responsible for the development of type IV pili nanomotors [104, 105], and the *gvp* genes are involved in the synthesis of gas vesicles that increase cell buoyancy [106–108]. Both type IV pili and gas vesicles are needed for the gliding motility of hormogonia [105, 109]. Taken together, our results indicate that both early and late stages of motile hormogonia development are repressed in lichenized *Nostoc*.

The transcriptional downregulation signal was stronger in the water-saturated samples compared to the natural samples (Fig. 4 and Supplementary Fig. S8). However, genes essential for developing motile hormogonia such as *sigC* and *gvpA* were significantly downregulated in both conditions (*sigC*: water-saturated Log_2_FC = −3.56, *P* = 1.81 × 10^−9^, natural Log_2_FC = −2.60, *P* = 9.82 × 10^−8^; *gvpA*: water-saturated Log_2_FC = −8.57, *P* = 1.04 × 10^−7^, natural Log_2_FC = −4.57, *P* = 1.51 × 10^−6^; Fig. 4 and Supplementary Fig. S8). Mutant strains of *Nostoc punctiforme* ATCC 29133 lacking a functional copy of *gvpA* are non-motile [109], and mutants lacking a functional copy of *sigC* do not even develop hormogonia [104]. Therefore, the transcriptional profiles indicate that the development of motile hormogonia is repressed in lichenized *Nostoc* in both natural and water-saturated conditions. This is in agreement with anatomical observations where lichenized *Nostoc* occur as vegetative filaments with smooth cell surfaces (i.e. non-piliated) and heterocysts [68, 87]. As far as we know, there are no reports of *Nostoc* hormogonia in cyanolichen thalli.

Our results raise the intriguing possibility that the cyanolichen fungus secretes hormogonia repressing factors (HRF), which would mirror HRF secretion by plant hosts after colonization by symbiotic *Nostoc* [8]*.* Although the precise composition of the HRF secreted by plants is unknown, it is suspected to include soluble sugars [8, 110]. Indeed, previous studies [111] found that sucrose and sucralose suppressed hormogonia development in *N. punctiforme* ATCC 29133. They also showed that high concentrations of both sucrose and sucralose induced a morphology where filaments were tightly coiled and surrounded by a polysaccharide sheath [111]. This is the same morphology displayed by *Nostoc* in the symbiotic plant tissues of *Anthoceros* sp. [111], cycads [112], and *Gunnera* [113], as well as by symbiotic *Nostoc* in cyanolichen thalli [87, 114, 115]. In symbiosis with plants, it is intuitive to think of sucrose (or a similar disaccharide) as a signaling molecule that triggers these developmental changes because sugars flow from the plant host to the *Nostoc* symbionts [8, 19]. However, the situation is reversed in the cyanolichen symbiosis, where sugars flow from *Nostoc* to the lichen fungus [13] (Fig. 1B). Moreover, none of the isotope tracing studies with lichenized *Nostoc* found an accumulation of fixed carbon into sucrose or another disaccharide [24, 26]. Therefore, it is unlikely that the lichen fungus secretes a sugar molecule that could elicit these developmental changes in lichenized *Nostoc*.

Our study revealed key genomic and expression changes that are linked with *Nostoc*’s metabolic role in the cyanolichen symbiosis. Carbon metabolism in lichenized *Nostoc* is characterized by transcriptional upregulation of genes for photosynthetic carbon fixation (Fig. 1A, Supplementary Figs S3A and S4), a D-enzyme involved in non-canonical degradation of glycogen, and the GlcP glucose permease (Fig. 1B and C, and Supplementary Fig. S3B and C). These genes are likely involved in glucose transfer from *Nostoc* to the cyanolichen fungus. We also found that the transfer of ammonium from *Nostoc* to the cyanolichen fungus is facilitated by two factors: (i) transcriptional downregulation of glutamine synthetase (Fig. 2B and Supplementary Fig. S6), the key enzyme responsible for ammonium assimilation in *Nostoc* (Fig. 2A); and (ii) frequent losses of a putative high-affinity ammonium permease (Fig. 3), which likely reduces *Nostoc*’**s capacity to recapture leaked ammonium. In addition, lichenized *Nostoc* commonly harbor both Mo- and V-Nases (Fig. 3). This is likely advantageous in high-latitude ecosystems where molybdenum can limit nitrogen fixation, temperatures are lower, and nitrogen is often limiting. Finally, we found that the development of motile hormogonia is repressed in lichenized *Nostoc* (Fig. 4 and Supplementary Fig. S8). Future studies should investigate potential signaling mechanisms that trigger the metabolic and developmental rewiring that we observed in lichenized *Nostoc*.

## Supplementary Material

supplementary_material_wraf166

supplementary_tables_isme_wraf166

## Data Availability

All newly generated sequence data were deposited in GenBank under BioProject accession PRJNA1223958.
