# Supplementary material for: Central metabolism and development are rewired in lichenized cyanobacteria
Source: ISME J. 2025 Aug 6;19(1):wraf166. doi: 10.1093/ismejo/wraf166 (PMC12416817; doi:10.1093/ismejo/wraf166)
Supplement: supplementary_material_wraf166 [file supplementary_material_wraf166.pdf]

Supplementary Materials

Central metabolism and development are rewired in lichenized cyanobacteria

Diego Garfias-Gallegos[1*], Carlos J. Pardo-De la Hoz[1*], Diane L. Haughland[2], Nicolas Magain[3], Blanka Aguero[1], Jolanta Miadlikowska[1], François Lutzoni[1†].

[1]Department of Biology, Duke University, Durham, NC 27708, USA.

[2]Department of Renewable Resources, Faculty of Agricultural, Life & Environmental Sciences, University of Alberta, Edmonton, AB T6G 2H1, Canada.

[3]Evolution and Conservation Biology, InBioS Research Center, Université de Liège, Liège 4000, Belgium.

[*]These authors contributed equally to this work.

[†]Corresponding author. Email: francois.lutzoni@duke.edu

Supplementary Methods

Natural and water-saturated sample collection

For the natural set, we first located three thalli from each of four different *Peltigera* morphospecies: *Peltigera canina s. lat.* (c1–c3), *Peltigera elisabethae* (e1–e3), *Peltigera malacea* (m1–m3), and *Peltigera appalachiensis* (n1–n3). The 12 thalli were < 100 m of each other and grew on feathermosses (Supplementary Fig. S1A–S1C). For each thallus, we sampled (i) a ~1 cm2 piece from a lobe tip of the lichen thallus, and (ii) feathermosses from four points immediately adjacent to the lichen thallus. Therefore, we collected a total of 12 lichen samples and 12 pooled feathermoss samples (Supplementary Table S2 and S3). The sampling was completed between 6:30 AM and 8:30 AM on July 20th, 2022. We did not collect climatic data during sampling, but the closest weather station (SLAVE LAKE RCS, ~47 km away, data from https://climate.weather.gc.ca/) recorded temperatures between 13.5 °C and 16 °C during the sampling period, and 3.2 mm of rain over the five days preceding sampling.

For the water-saturated set, we collected mats that included the lichen thalli sampled above and their feathermoss substrate and transported them to Edmonton, Alberta (2.5 h drive). The next day, we placed the mats outside under indirect natural light and sprayed them with water until water-saturated (e.g., Supplementary Fig. S1D). We then sampled the lichen thalli and feathermoss substrate in the same way as the natural set. This sampling was completed between 6:30 AM and 7:00 AM on July 21st, 2022.

Nucleic acid isolation and sequencing

We isolated metagenomic DNA from dried samples of the 12 lichen thalli and 12 feathermoss mats using an SDS protocol as described previously [1]. Metagenomic libraries were prepared with the KAPA HyperPrep Kit (Roche Sequencing Solutions, Pleasanton, CA, USA) following the manufacturer's instructions and sequenced on a Novaseq 6000 (Illumina) S1 flow cell (150 bp PE). We also sequenced the metagenomic DNA isolated from the 12 lichen samples in two R10.4.1 flow cells from Oxford Nanopore Technologies. We isolated RNA with the RNesay Plant Mini Kit (Qiagen, Hilden, Germany) following the manufacturer's instructions. Metatranscriptomic libraries were prepared with the ZymoSeq RiboFree Total RNA Library Kit (Zymo Research, Irvine, CA, USA) and sequenced on a Novaseq 6000 (Illumina) S-Prime flow cell (150 bp PE).

Custom database for kraken2-bracken

We generated a custom database to improve the resolution of taxonomic assignments inferred with kraken2-bracken [2]. We first downloaded 225 genomes of *Nostoc s. lat.* from public databases (Supplementary Table S1); 80,789 bacterial and archaeal genomes from the GTDB [3] database (V214 released in 2023-06-09); 2,537 fungal genomes from the MycoCosm [4] database (accessed in 2023-11-05); and 106 *Peltigera* MAGs [1]. Next, we use custom scripts to change the header of all the contigs in these sequences to have a compliant structure with the kraken2 database (kraken:taxid| NCBI taxon ID number). Finally, we used these trimmed sequences and the standard kraken2 database to create a kraken2-bracken database using parameters: -s 4 -k 49.

Metagenomic assembly and* Nostoc *genome binning

We used fastp v0.23 [5] to trim reads in a sliding window of 15 bp when < 80% bases had < Q18 PHRED score. After trimming, reads with < 100 bp were discarded. For the lichen metagenomic libraries, we classified reads from both long- and short-read datasets with our curated kraken2-bracken v2.1.3 [6] database, and extracted the reads classified as *Nostoc* from each library using kraken-tools v1.2 [7]. We then used Unicycler v0.5.1 [8] to carry out hybrid assemblies with both long- and short-reads of *Nostoc*. We recovered 13 *Nostoc* metagenome-assembled genomes (MAGs) from the 12 lichen libraries.

For the 12 feathermoss libraries, we used two different approaches to recover *Nostoc* MAGs. First, we extracted reads classified as *Nostoc* with kraken-tools v1.2 and assembled them using SPAdes v4.0.0 [9]. This generated three *Nostoc* MAGs: e2_top_contigs, m1_top_contigs, and n2_top_contigs (Supplementary Table S1). Second, we assembled the complete metagenomes of the feathermoss libraries using the --meta option in SPAdes v4.0.0 [10] and binned the assembled contigs with VAMB v4.1.3 [11]. This generated three additional *Nostoc* MAGs: c2_topC1511, m1_topC1290, and n2_topCmix (Supplementary Table S1).

To remove contaminant sequences, we classified the contigs in all 19 *Nostoc* MAGs (13 from lichen libraries and six from feathermoss libraries) with MMseqs2 v15.0.0 [12] using the UniRef90 and UniProtKB databases and discarded contigs not classified as *Nostoc*. The quality of the MAGs was assessed with CheckM v1.2.3 [13], QUAST v5.2.0 [14], and BUSCO v5.0.0 [15] coupled with the nostocales_odb10 database [16]. Based on this, we retained 12 *Nostoc* MAGs from the lichen libraries (one from each lichen thallus) and six MAGs from the

feathermoss libraries with > 90% completeness and < 5% contamination (Supplementary Table S1).

Finally, we classified the contigs from the *Nostoc* MAGs into chromosome and plasmid origin using PlasX [17]. We used a classification threshold of 0.1 and reported the cumulative length of sequence classified as plasmid for *Nostoc* genome in Supplementary Table S1.

Genome annotation with profile HMMs of target genes

We generated profile hidden Markov models (HMMs) of each target gene and then used those profile HMMs to search for the genes in our sample of 246 genomes [18]. To generate the profile HMMs, we retrieved a minimum of five nucleotide sequences of each target gene from genomes of nostocalean cyanobacteria available in the NCBI and UniProt databases. Next, we aligned each set of sequences using MAFFT v7.475 [19] with the –auto flag. We used these alignments as input for HMMER v3.4 [18] to produce profile HMMs. We then used custom scripts that integrated HMMER and SeqKit v2.8 [20] to search and extract the nucleotide sequences of the target genes from the 246 genomes in our sampling. We visualized the genome content across strains using heatmaps generated with the R package ComplexHeatmap v2.20.0 [21].

We used one of two possible criteria to select e-value thresholds for the HMMER searches. (i) If the profile HMM included sequences from *Nostoc punctiforme* ATCC 29133 or *Nostoc flagelliforme* CCNUN1 (which were part of our sampling of *Nostoc s. lat.* genomes), we used the e-value of the HMMER hit obtained for either of those two genomes as a maximum threshold for the searches in the rest of the genomes. (ii) If the profile HMM did not include sequences from *Nostoc punctiforme* ATCC 29133 or *Nostoc flagelliforme* CCNUN1, we conducted a BLASTn search of the HMMER hits against the NCBI nucleotide database. Then, we filtered the BLASTn results to retain hits with >80% similarity to sequences with the same annotation as the profile HMM in a set of reference genomes (*Anabaena* sp. PCC 7120, *Anabaena variabilis* ATCC 29413, *Synechocystis* sp. PCC 6803, or *Synechococcus elongatus* PCC 7942). Finally, we selected the highest e-value among the HMMER hits that fulfilled these similarity and annotation criteria after the BLASTn search to use it as a maximum threshold for the searches in the rest of the genomes. All e-values we used for each target gene are in Supplementary Table S4.

Screening* **Nostoc** *genomes for glucose transporters

We used two strategies to search for glucose transporter genes that could be involved in glucose transfer from *Nostoc* to the cyanolichen fungus. First, we conducted HMMER and BLASTp searches of the *Nostoc* genomes using the amino acid sequences of annotated glucose transporters from the Transporter Classification Database (TCDB) [22]. Second, we generated a custom HMM profile and conducted a targeted HMMER search of *glcP* homologs, a putative glucose permease found in *Nostoc punctiforme* and *Synechocystis* sp. PCC6803 [23]. Both the TCDB and the *glcP* strategy recovered the same hits for every lichenized genome (Supplementary Table S5).

Mapping of* Nostoc *reads from metatranscriptomic libraries

To include only genes shared across *Nostoc* genomes of both lichenized and feathermoss-associated datasets, we retrieved the genes present in at least 75% of the genomic data for each *Nostoc* lifestyle (i.e., lichenized [nine out of 12 libraries], and feathermoss-associated [five out of six libraries]). Then, we kept genes that passed this threshold in both lichenized and feathermoss-associated datasets (298 out of 514, Supplementary Table S4). The *Nostoc* reads from each lichen metatranscriptomic library were mapped to the 298 selected gene sequences of the *Nostoc* MAG assembled from the corresponding lichen specimen. For example, the *Nostoc* reads from the c1 metatranscriptomic library were mapped to annotated gene sequences from the *Nostoc* MAG assembled from the c1 metagenomic library. For the *Nostoc* associated with feathermosses, we first built a hybrid reference by combining the 298 genes annotated in the six *Nostoc* MAGs obtained from the feathermoss metagenomic libraries (five MAGs from *Nostoc* section 2.4 and one MAG from *Nostoc* section 3.7; Supplementary Fig. S2). We then deduplicated the combined gene sequences using the dedupe.sh script part of BBTools v37.62.0 [24] with options minidentity=98 and e=10 (maximum indel number). The *Nostoc* reads extracted from each feathermoss metatranscriptomic library were mapped to this hybrid, deduplicated set of reference genes. The use of this hybrid reference allowed us to capture the gene expression profile of the community of *Nostoc* associated with feathermosses rather than a particular strain.

Phylogeny of ammonium permeases

We inferred a phylogeny of ammonium permeases that included (i) 386 protein sequences derived from our targeted annotation (*amt1, amt4, and amtB* genes; Supplementary Table S5) of 243 genomes of *Nostoc s. lat.,* and (ii) 57 protein sequences that represent the diversity of the AMT/MEP/Rh family of ammonium permeases (Supplementary Table S6) [25]. We aligned the

amino acid sequences using MAFFT v7.475 with the DASH option to incorporate protein structural information and the G-INS-i iterative refinement algorithm [26]. We then searched for the best fitting model of amino acid substitution considering site-heterogeneous models. LG+C50+F was selected as the optimal model using the Bayesian information criterium and we used it to infer a maximum likelihood phylogeny with 1,000 UFBoot2 pseudoreplicates in IQ-Tree v1.6.12 [27,28]. The tree was visualized with the R package *ggtree* v.3.4.4 [29].

Quantifying association between **amt1** *presence and lichenized lifestyle*

We used a phylogenetic generalized linear model to explicitly quantify the relationship between the lichenized lifestyle and the presence/absence of the *amt1* gene while accounting for the underlying phylogeny. Lichenized versus non-lichenized refer to *Nostoc* lifestyles at the time of our sampling, exclusively. We fitted a phylogenetic logistic regression of the form *amt1* ~ lifestyle, where the lifestyle variable was encoded as either lichenized or non-lichenized. We used the phyloglm function from the phylolm v2.6.5 R package for model fitting [30].

Estimation of false negative rate of V-Nase gene detection

V-Nase genes are often carried in plasmids [31]. Therefore, searching V-Nase genes in MAGs can yield false negative detection if the MAGs are missing plasmid sequences. To estimate the rate of false negative detection of V-Nase genes in MAGs, we searched the *Nostoc* V-Nase genes in the full metagenome assemblies from cyanolichens, which were available to us, and compared the detections with those obtained from the *Nostoc* MAGs binned from the same metagenome assemblies (Supplementary Table S5). Then we calculated a false negative rate (FNR) as:

$$FNR = \frac{N_{metagenome\ V-Nas} \ -N_{MAG\ V-Nase}}{N_{metagenome\ V-Nase}} * 100\% = \frac{109-}{109} * 100\% = 11\%.$$

This FNR is especially relevant for the *Nostoc* symbionts of cycads, because 90% (38/42) of these genomes are MAGs (Supplementary Table S1). If we account for the FNR for MAGs, the percentage of cycad-symbiont genomes with V-Nase genes would increase from 7% (3/42 genomes) to 16% (7/42, i.e., four genomes with false negative V-Nase gene detections), which is still substantially lower than in genomes of lichenized *Nostoc* (66% in MAGs, or 76% after considering V-Nase detection across the entire metagenome assemblies).

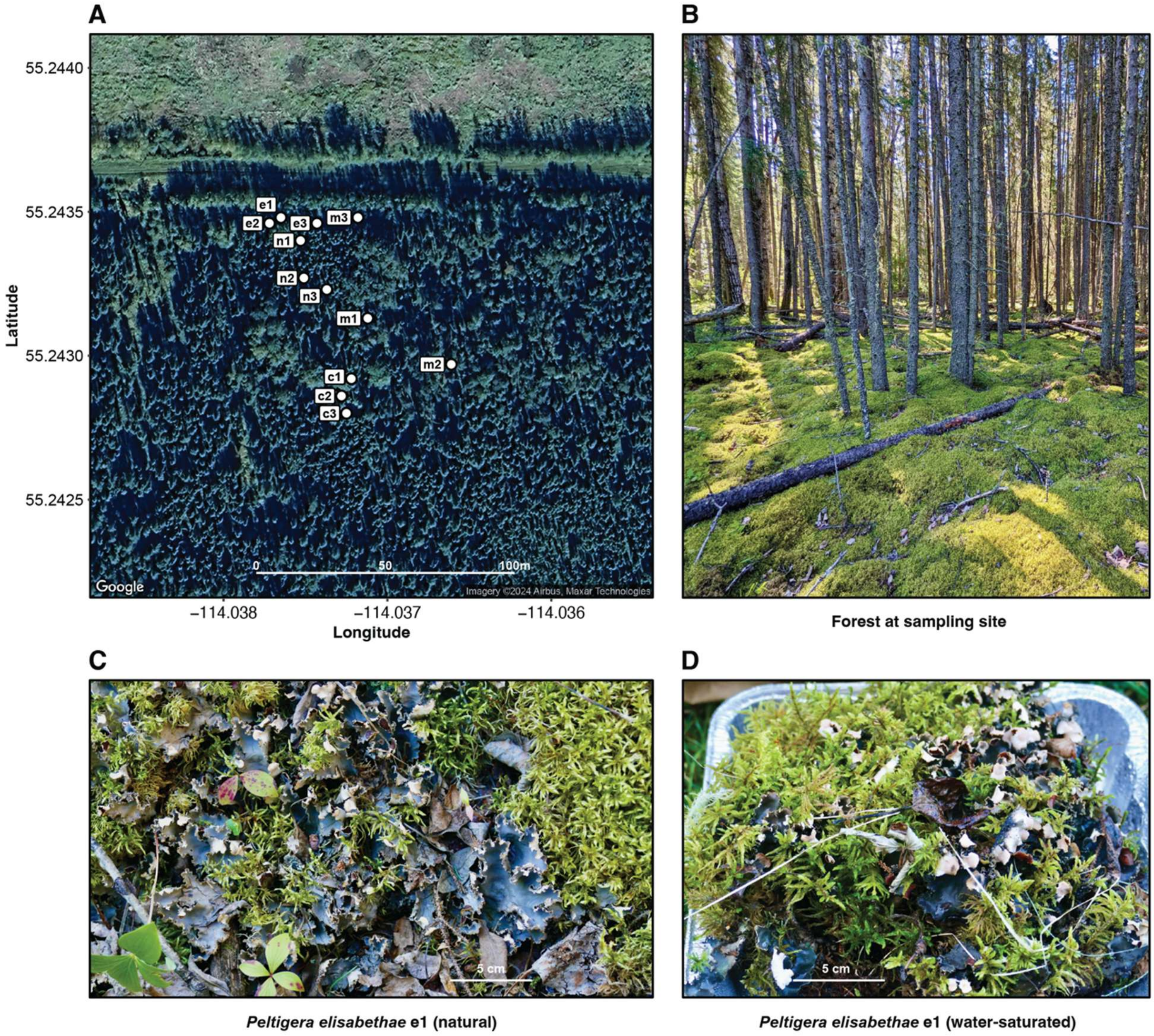

Supplementary Figure S1. Sampling of cyanolichen and adjacent feathermoss for metatranscriptomic and metagenomic sequencing. **A** Location of 12 thalli (Supplementary Table S2) from four different *Peltigera* morphospecies (*Peltigera canina s. lat.* [c1–c3], *Peltigera elisabethae* [e1–e3], *Peltigera malacea* [m1–m3], and *Peltigera appalachiensis* [n1–n3]) at sampling site in Alberta, Canada. **B** Forest at sampling site dominated by *Picea mariana* and ground cover dominated by feathermosses. **C** and **D** show examples of a cyanolichen specimen (*Peltigera elisabethae* e1) growing on feathermosses in natural (**C**) and saturated (**D**) water content conditions prior to our sampling for metatranscriptomic sequencing.

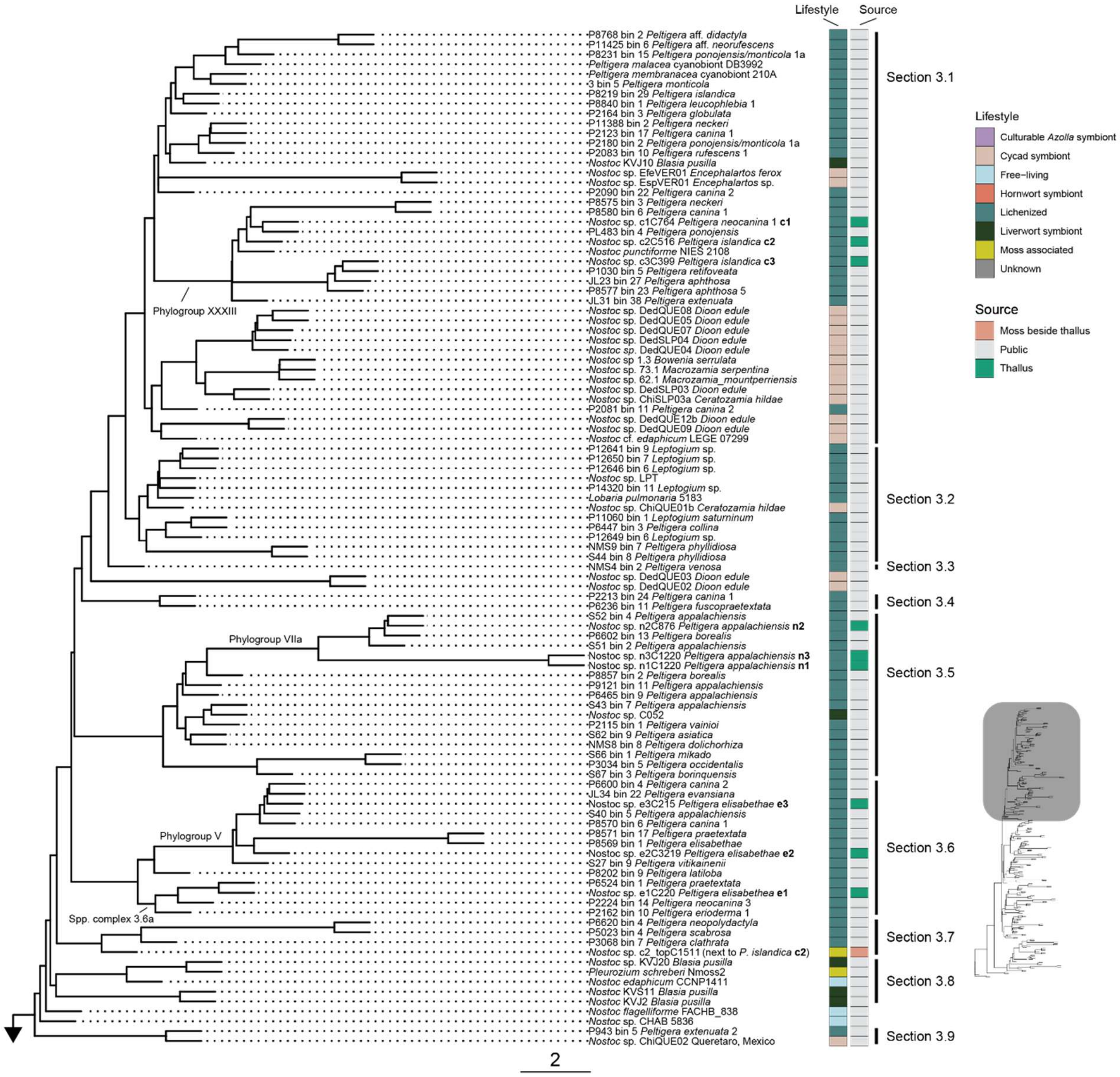

Supplementary Figure S2. Species tree of 243 *Nostoc s. lat.* genomes and three outgroup taxa included in the study. The tree was inferred with weighted-ASTRAL using 1,517 genes. Branch lengths represent coalescent units. Genome metadata is in Supplementary Table S1. The source column to the right of the tree indicates whether a genome was sequenced in this study ("Moss besides thallus" or "Thallus") or retrieved from public databases ("Public"). Phylogenetic sections of *Nostoc* to the right of the Lifestyle and Source columns are as described previously [1].

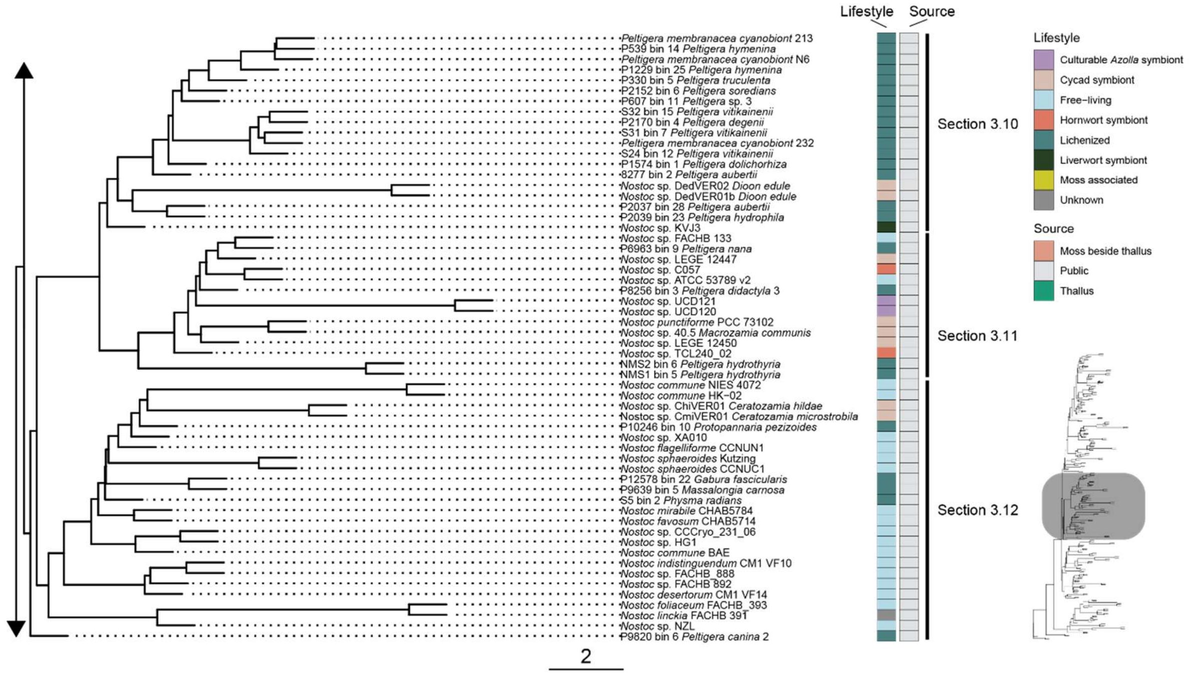

Supplementary Figure S2. (continued)

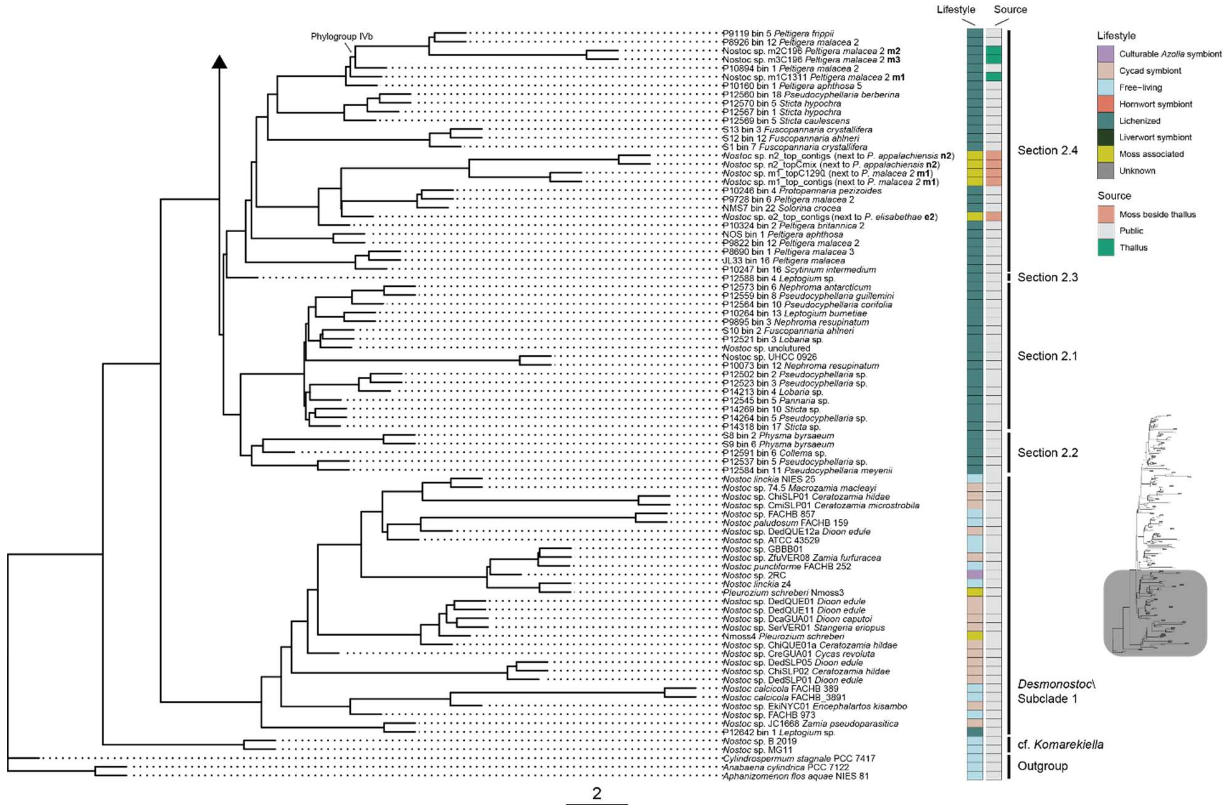

Supplementary Figure S2. (continued)

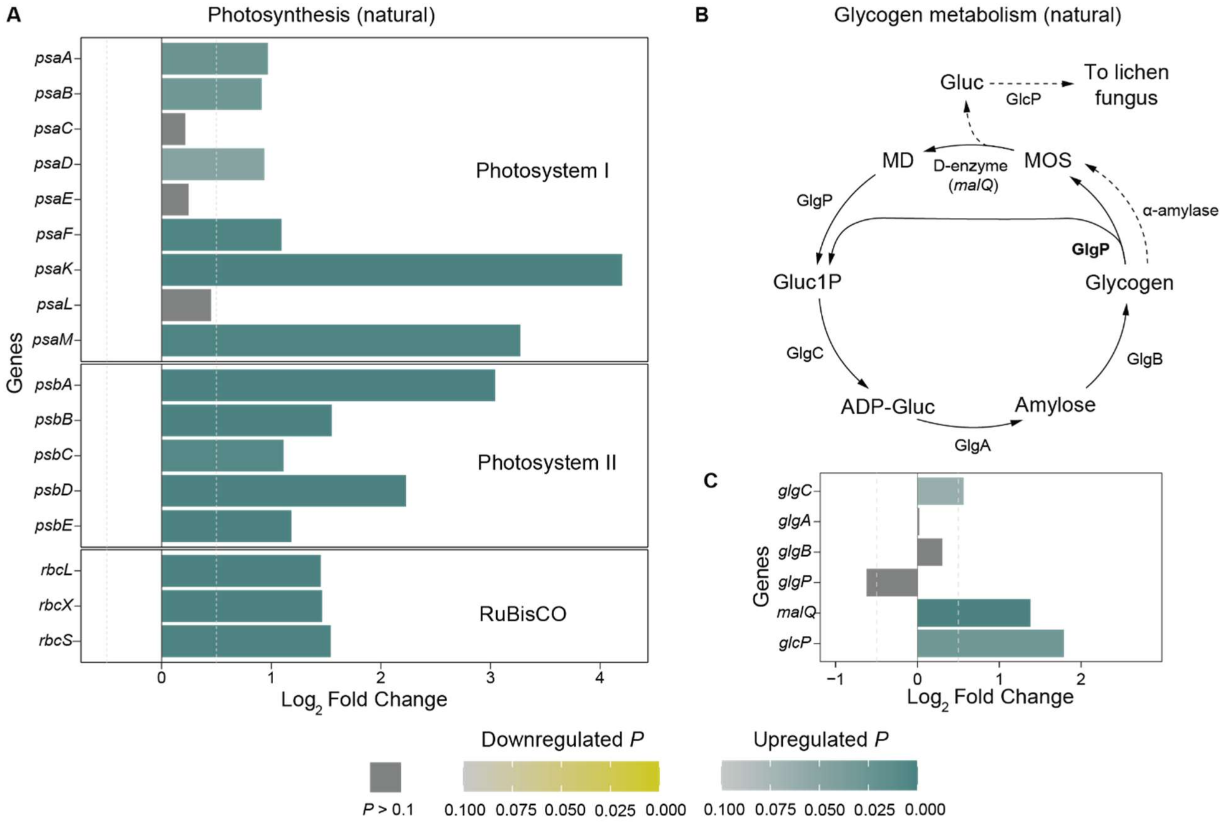

Supplementary Figure S3. Photosynthesis and putative symbiotic glycogen degradation are upregulated in lichenized *Nostoc* in natural samples. **A** Differential expression of genes that encode three key protein complexes involved in photosynthetic carbon fixation. **B** Glycogen synthesis and degradation in cyanobacteria. Solid arrows show the canonical pathway (https://www.genome.jp/pathway/hsa00500) [32]. Dashed arrows show the proposed mechanism of glycogen degradation that leads to glucose released by lichenized *Nostoc* [33]. **C** Differential expression of glycogen metabolism genes. Log_2 Fold Change and adjusted *P* values were estimated with DESeq2 based on 12 lichenized and six adjacent moss-associated *Nostoc* RNA-seq libraries from samples obtained from cyanolichens and their moss substrates under natural conditions. Gene descriptions are in Supplementary Table S4. Gluc1P: glucose-1-phosphate; ADP-Gluc: adenosine diphosphate glucose; MOS: maltooligosaccharides; MD: maltodextrins; GlgC: glucose-1-phosphate adenylyltransferase; GlgA: glycogen synthase; GlgB: glucan-branching enzyme; GlgP: glycogen phosphorylase; *malQ*: putative D-enzyme; GlcP: glucose permease.

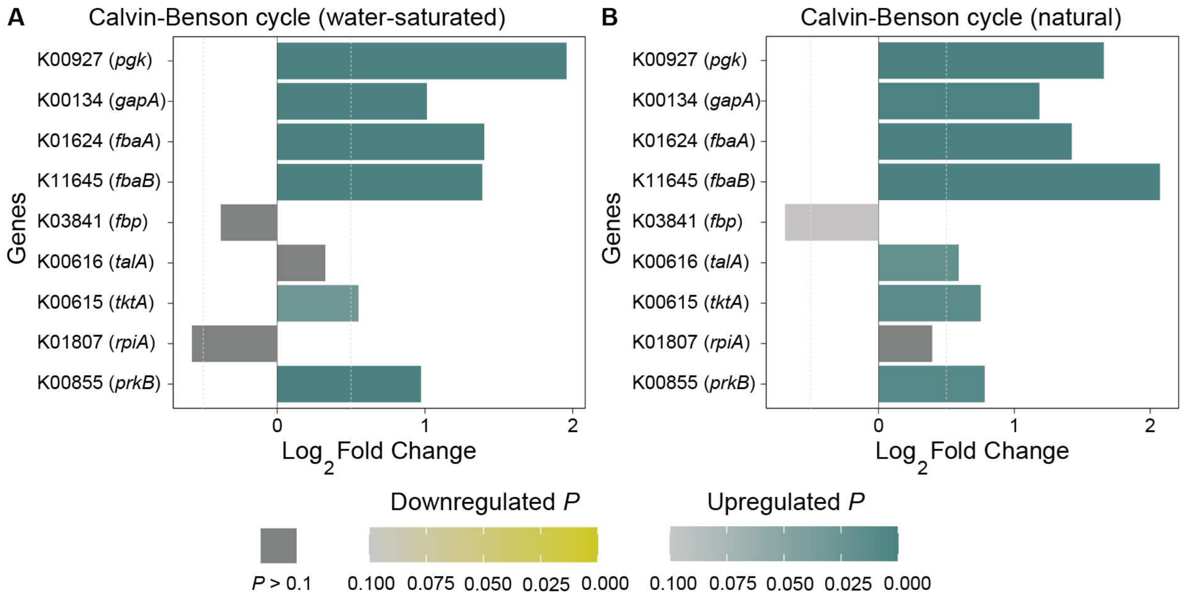

Supplementary Figure S4. Genes of the Calvin-Benson cycle are upregulated in lichenized *Nostoc*. Both panels show differential expression as Log_2 Fold Change and adjusted *P* values estimated with DESeq2. Estimates in panel **A** are based on 12 lichenized and seven moss-associated *Nostoc* RNA-seq libraries from water-saturated samples. Estimates in panel **B** are based on 12 lichenized and six moss-associated *Nostoc* RNA-seq libraries obtained from cyanolichens and their moss substrates under natural conditions. Gene labels include both KEGG entry codes and gene abbreviations.

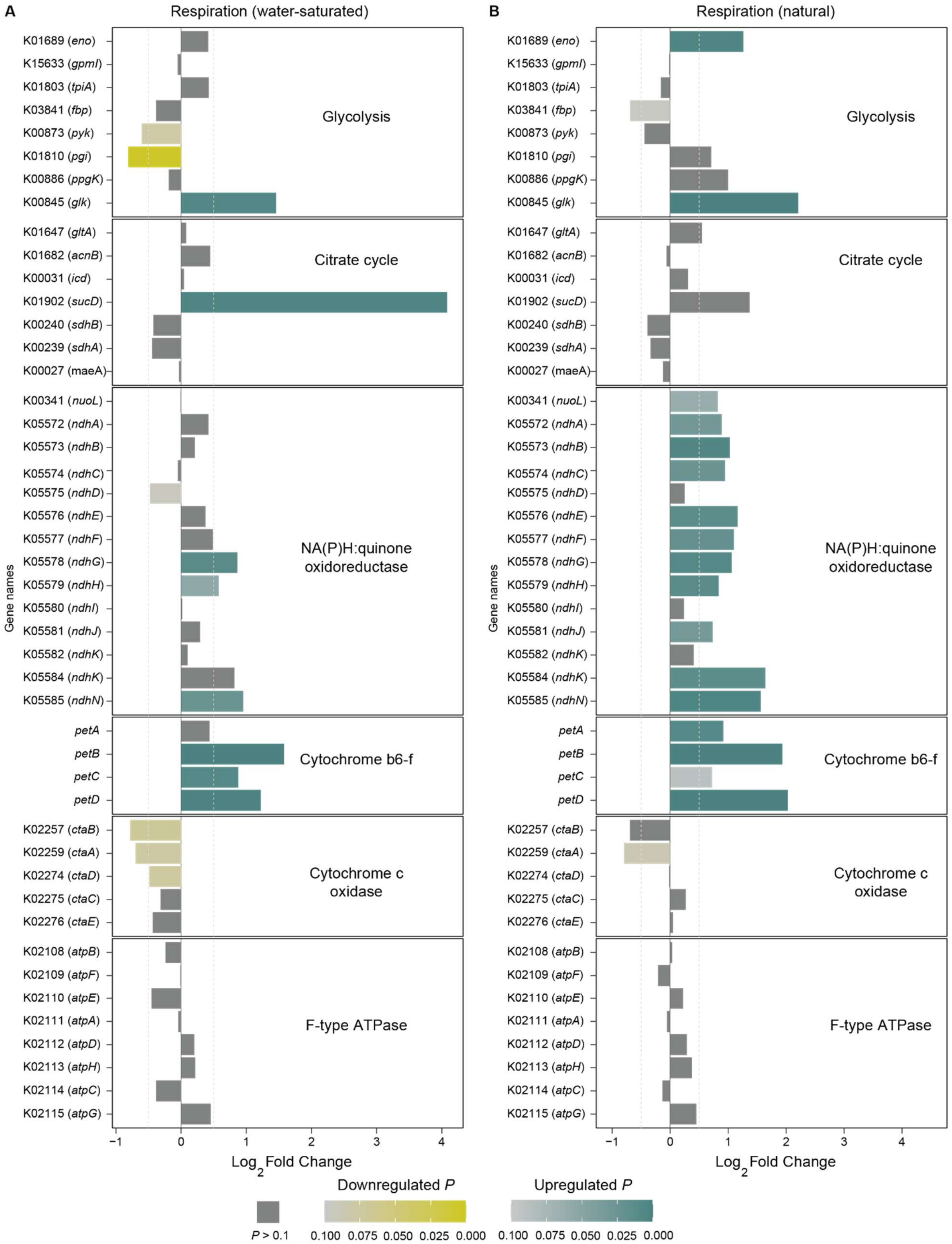

A
Respiration (water-saturated)
B
Respiration (natural)
Glycolysis
Citrate cycle
NA(P)H:quinone oxidoreductase
Cytochrome b6-f
Cytochrome c oxidase
F-type ATPase
Gene names
K01689 (eno)
K15633 (gpmI)
K01803 (tpiA)
K03841 (fbp)
K00873 (pyk)
K01810 (pgi)
K00886 (ppgK)
K00845 (glk)
K01647 (gltA)
K01682 (acnB)
K00031 (icd)
K01902 (sucD)
K00240 (sdhB)
K00239 (sdhA)
K00027 (maeA)
K00341 (nuoL)
K05572 (ndhA)
K05573 (ndhB)
K05574 (ndhC)
K05575 (ndhD)
K05576 (ndhE)
K05577 (ndhF)
K05578 (ndhG)
K05579 (ndhH)
K05580 (ndhI)
K05581 (ndhJ)
K05582 (ndhK)
K05584 (ndhK)
K05585 (ndhN)
petA
petB
petC
petD
K02257 (ctaB)
K02259 (ctaA)
K02274 (ctaD)
K02275 (ctaC)
K02276 (ctaE)
K02108 (atpB)
K02109 (atpF)
K02110 (atpE)
K02111 (atpA)
K02112 (atpD)
K02113 (atpH)
K02114 (atpC)
K02115 (atpG)
−1 0 1 2 3 4
Log2 Fold Change
Downregulated P
Upregulated P
P > 0.1
0.100 0.075 0.050 0.025 0.000
0.100 0.075 0.050 0.025 0.000

Supplementary Figure S5. Most genes involved exclusively in cellular respiration pathways (i.e., glycolysis, citrate cycle, and oxidative phosphorylation) are not differentially expressed in lichenized *Nostoc* compared to *Nostoc* associated with feathermosses. Note that the NAD(P)H:quinone oxidoreductase and the cytochrome b6-f also participate in photosynthetic electron transport as well as oxidative phosphorylation during respiration. Both panels show differential expression as Log_2 Fold Change and adjusted *P* values estimated with DESeq2. Estimates in panel **A** are based on 12 lichenized and seven moss-associated *Nostoc* RNA-seq libraries from samples saturated with water. Estimates in panel **B** are based on 12 lichenized and six moss-associated *Nostoc* RNA-seq libraries from samples under natural conditions. Gene labels include both KEGG entry codes and gene abbreviations.

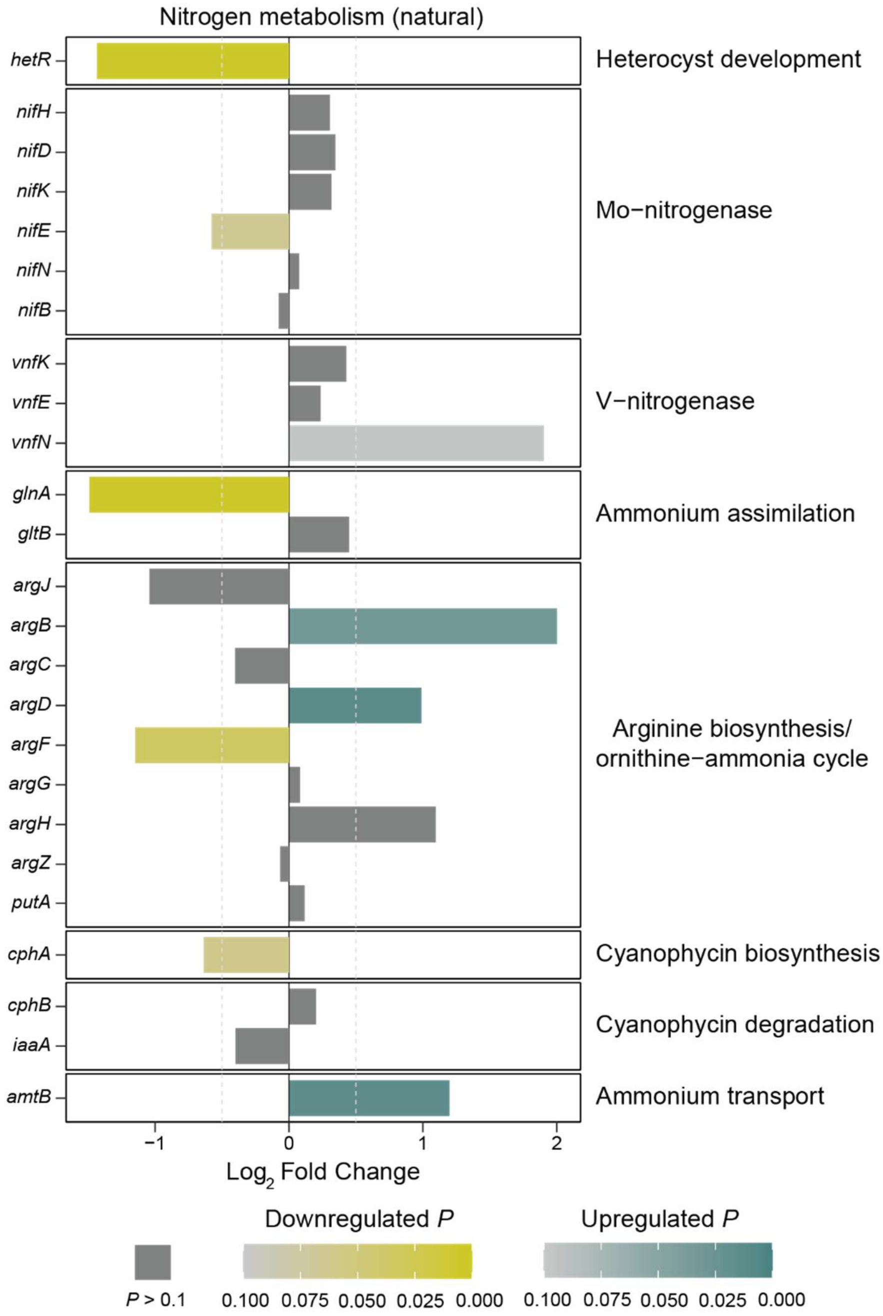

Supplementary Figure S6. Downregulated heterocyst development and ammonium assimilation is associated with signatures of growth under nitrogen deficiency conditions in lichenized *Nostoc* in natural samples. Differential expression of genes involved in dinitrogen assimilation. Log_2 Fold Change and adjusted *P* values were estimated with DESeq2 based on 12 lichenized and six moss-associated *Nostoc* RNA-seq libraries from natural samples. Gene descriptions are in Supplementary Table S4.

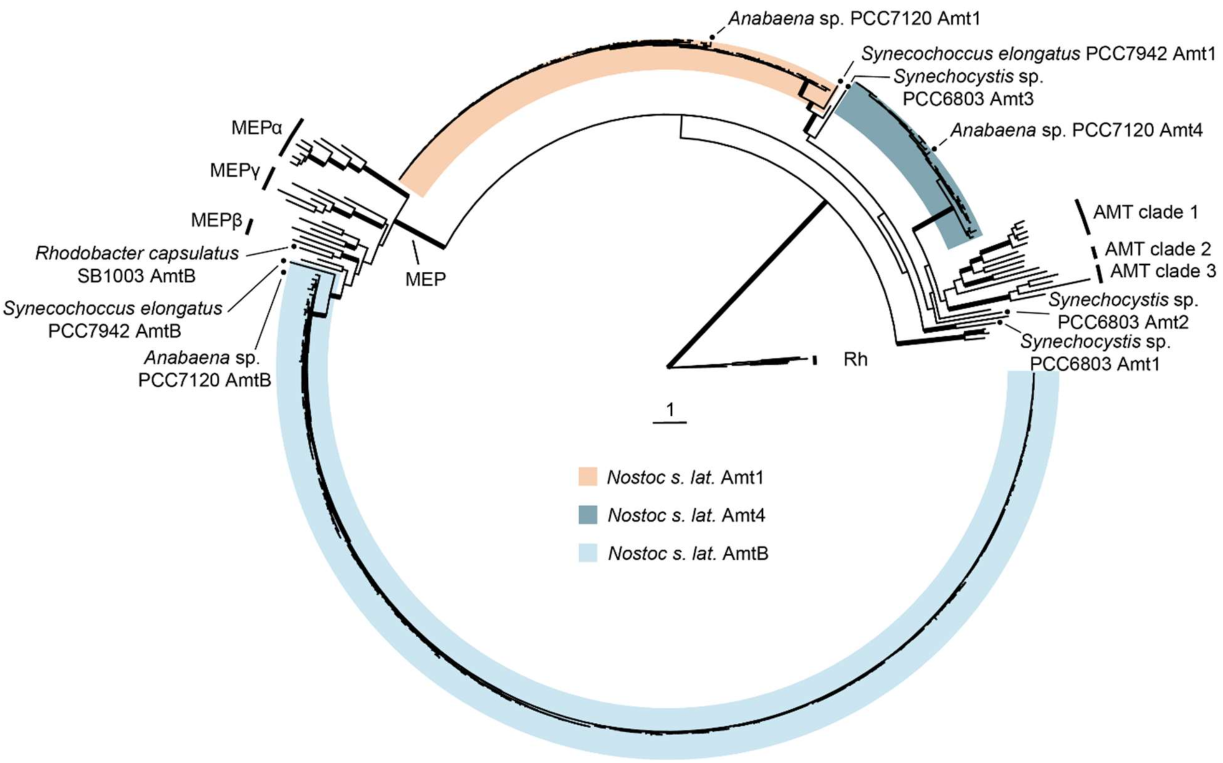

Supplementary Figure S7. Genomes of *Nostoc s. lat.* encode up to three types of putative ammonium permeases from the AMT/MEP/Rh family. Maximum likelihood protein phylogeny of the AMT/MEP/Rh family. Reference sequences and clade delimitations follow previous observations [25] (Supplementary Table S6). Thick branches indicate UFBoot2 support > 95%. Branch lengths represent the average number of amino acid substitutions per site.

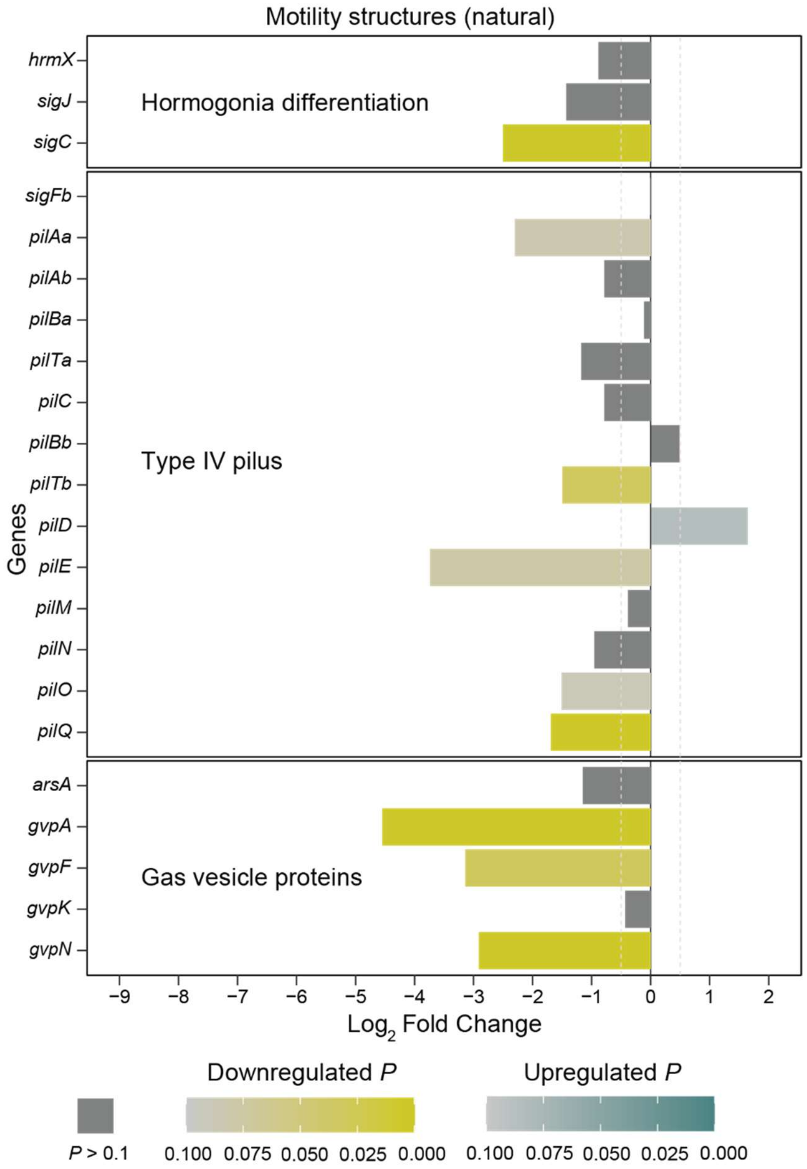

Supplementary Figure S8. Development of motility structures is downregulated in lichenized *Nostoc* in natural samples. Differential expression of structural and regulatory genes for the development of motility structures. Log_2 Fold Change and adjusted *P* values were estimated with DESeq2 based on 12 lichenized and six moss-associated *Nostoc* RNA-seq libraries from natural samples. Gene descriptions are in Supplementary Table S4.

References

1. Pardo-De la Hoz CJ, Haughland DL, Thauvette D *et al.* Rapid radiations outweigh reticulations during the evolution of a 750-million-year-old lineage of cyanobacteria. *In Review*.

2. Lu J, Breitwieser FP, Thielen P *et al.* Bracken: Estimating species abundance in metagenomics data. *PeerJ Comput Sci* 2017;**3**:e104.

3. Parks DH, Chuvochina M, Rinke C *et al.* GTDB: An ongoing census of bacterial and archaeal diversity through a phylogenetically consistent, rank normalized and complete genome-based taxonomy. *Nucleic Acids Res* 2022;**50**:D785–94.

4. Ahrendt SR, Mondo SJ, Haridas S *et al.* MycoCosm, the JGI's fungal genome portal for comparative genomic and multiomics data analyses. In: Martin F, Uroz S (eds.). *Microbial Env Genomics*. New York, NY: Springer US, 2023, 271–91.

5. Chen S, Zhou Y, Chen Y *et al.* fastp: An ultra-fast all-in-one FASTQ preprocessor. *Bioinformatics* 2018;**34**:i884–90.

6. Wood DE, Lu J, Langmead B. Improved metagenomic analysis with Kraken 2. *Genome Biol* 2019;**20**:257.

7. Lu J, Rincon N, Wood DE *et al.* Metagenome analysis using the Kraken software suite. *Nat Protoc* 2022;**17**:2815–39.

8. Wick RR, Judd LM, Gorrie CL *et al.* Unicycler: Resolving bacterial genome assemblies from short and long sequencing reads. *PLoS Comput Biol* 2017;**13**:1–22.

9. Bankevich A, Nurk S, Antipov D *et al.* SPAdes: A new genome assembly algorithm and its applications to single-cell sequencing. *J Comput Biol* 2012;**19**:455–77.

10. Nurk S, Meleshko D, Korobeynikov A *et al.* metaSPAdes: A new versatile metagenomic assembler. *Genome Res* 2017;**27**:824–34.

11. Nissen JN, Johansen J, Allesøe RL *et al.* Improved metagenome binning and assembly using deep variational autoencoders. *Nat Biotechnol* 2021;**39**:555–60.

12. Steinegger M, Söding J. MMseqs2 enables sensitive protein sequence searching for the analysis of massive data sets. *Nat Biotechnol* 2017;**35**:1026–8.

13. Parks DH, Imelfort M, Skennerton CT *et al.* CheckM: Assessing the quality of microbial genomes recovered from isolates, single cells, and metagenomes. *Genome Res* 2015;**25**:1043–55.

14. Gurevich A, Saveliev V, Vyahhi N *et al.* QUAST: Quality assessment tool for genome assemblies. *Bioinformatics* 2013;**29**:1072–5.

15. Simão FA, Waterhouse RM, Ioannidis P *et al.* BUSCO: Assessing genome assembly and annotation completeness with single-copy orthologs. *Bioinformatics* 2015;**31**:3210–2.

16. Kriventseva EV, Kuznetsov D, Tegenfeldt F *et al.* OrthoDB v10: Sampling the diversity of animal, plant, fungal, protist, bacterial and viral genomes for evolutionary and functional annotations of orthologs. *Nucleic Acids Res* 2019;**47**:D807–11.

17. Yu MK, Fogarty EC, & Eren AM. Diverse plasmid systems and their ecology across human gut metagenomes revealed by PlasX and MobMess. *Nature Microbiol* 2024, **9**(3), 830-847.

18. Eddy SR. Accelerated profile HMM searches. *PLOS Comput Biol* 2011;**7**:e1002195.

19. Katoh K, Standley DM. MAFFT Multiple sequence alignment software version 7: Improvements in performance and usability. *Mol Biol Evol* 2013;**30**:772–80.

20. Shen W, Le S, Li Y *et al.* SeqKit: A cross-platform and ultrafast toolkit for FASTA/Q file manipulation. *PLOS ONE* 2016;**11**:e0163962.

21. Gu Z, Eils R, Schlesner M. Complex heatmaps reveal patterns and correlations in multidimensional genomic data. *Bioinformatics* 2016;**32**:2847–9.

22. Saier MH, Reddy VS, Moreno-Hagelsieb *et al.* The transporter classification database (TCDB): 2021 update. *Nucleic Acids Res* 2021;**49**(D1), D461–D467. https://doi.org/10.1093/nar/gkaa1004

23. Ekman M, Picossi S, *et al.* A *Nostoc punctiforme* sugar transporter necessary to establish a cyanobacterium-plant symbiosis. *Plant Physiol* 2013;**161**(4), 1984–1992. https://doi.org/10.1104/pp.112.213116

24. Bushnell B, Rood J, Singer E. BBMerge – Accurate paired shotgun read merging via overlap. *PLOS ONE* 2017;**12**:e0185056.

25. McDonald TR, Ward JM. Evolution of electrogenic ammonium transporters (AMTs). *Front Plant Sci* 2016;**7**, DOI: 10.3389/fpls.2016.00352.

26. Rozewicki J, Li S, Amada KM *et al.* MAFFT-DASH: Integrated protein sequence and structural alignment. *Nucleic Acids Res* 2019;**47**:W5–10.

27. Hoang DT, Chernomor O, von Haeseler A *et al.* UFBoot2: Improving the ultrafast bootstrap approximation. *Mol Biol Evol* 2018;**35**:518–22.

28. Nguyen LT, Schmidt HA, Von Haeseler A *et al.* IQ-TREE: A fast and effective stochastic algorithm for estimating maximum-likelihood phylogenies. *Mol Biol Evol* 2015;**32**:268–74.

29. Yu G, Smith DK, Zhu H *et al.* ggtree: An r package for visualization and annotation of phylogenetic trees with their covariates and other associated data. *Methods Ecol Evol* 2017;**8**:28–36.

30. Tung Ho LS, & Ané C. A linear-time algorithm for Gaussian and non-Gaussian trait evolution models. *Sys Bio* 2014, **63**(3), 397–408. https://doi.org/10.1093/sysbio/syu005

31. Nelson JM, Hauser DA, Gudiño JA *et al.* Complete genomes of symbiotic cyanobacteria clarify the evolution of vanadium-nitrogenase. *Genome Biol Evol* 2019;**11**:1959–64.

32. Shinde S, Zhang X, Singapuri SP *et al.* Glycogen metabolism supports photosynthesis start through the oxidative pentose phosphate pathway in cyanobacteria. *Plant Physiol* 2020;**182**:507–17.

33. Wastlhuber R, Loos E. Differences between cultured and freshly isolated cyanobiont from *Peltigera*—is there symbiosis-specific regulation of a glucose carrier? *The lichenologist* 1996;**28.**
